# Lipidomic signatures in *Octopus vulgaris* arm muscle reveal geographic variation along the Iberian Atlantic Coast

**DOI:** 10.1038/s41538-025-00520-w

**Published:** 2025-08-09

**Authors:** Felisa Rey, Luís Gaspar, Fernando Ricardo, Cristina Pita, Maria do Rosário Domingues, Ricardo Calado

**Affiliations:** 1 CESAM - Centre for Environmental and Marine Studies, Department of Chemistry, University of Aveiro, Campus Universitário de Santiago, Aveiro, Portugal; 2 Mass Spectrometry Centre & LAQV-REQUIMTE, Department of Chemistry, University of Aveiro, Campus Universitário de Santiago, Aveiro, Portugal; 3 ECOMARE - Laboratory for Innovation and Sustainability of Marine Biological Resources, CESAM - Centre for Environmental and Marine Studies, Department of Biology, Campus Universitário de Santiago, University of Aveiro, Aveiro, Portugal; 4 CESAM - Centre for Environmental and Marine Studies, Department of Environment and Planning, University of Aveiro, Campus Universitário de Santiago, Aveiro, Portugal; 5 Institute of Marine Research (IIM-CSIC), Vigo, Pontevedra España

**Keywords:** Lipidomics, Lipids, Animal physiology

## Abstract

The common octopus (*Octopus vulgaris*) is a highly valued seafood species, whose flavour and palatability are often linked to its geographic origin. However, globalized supply chains have increased the risk of mislabelling. Lipid metabolism in marine organisms is shaped by environmental conditions, then lipidomics offers a promising tool for origin authentication. This study used HILIC-LC-MS&MS/MS to profile the polar lipidome of *O. vulgaris* arm muscle from three Iberian Atlantic landing sites: Ría de Arousa (Spain), Peniche, and Santa Luzia (Portugal). While total lipid content was consistent across sites, Peniche samples showed lower phospholipid levels, and Santa Luzia samples showed elevated LPE/PE and LPC/PC ratios, possibly reflecting stress or degradation. Ether-linked phospholipids and the sphingolipid CAEP were most abundant in Ría de Arousa, contributing to site discrimination. These findings demonstrate the potential of lipidomic profiling as a tool for seafood traceability, offering insights into environmental and dietary influences on lipid composition.

## Introduction

The common octopus (*Octopus vulgaris* Cuvier, 1797) is one of the most significant cephalopod fisheries in the European Union. These fisheries are both economically and ecologically important, particularly in regions such as Southern Europe and Northwest Africa^1^. Widely distributed across the Northeast Atlantic, Mediterranean, and Eastern Central Atlantic, *O. vulgaris* is particularly abundant along the Atlantic coasts of Portugal, Spain, Morocco, Mauritania, and Senegal^2^. In the Iberian Peninsula, it is one of the highest-revenue species, playing a particularly significant role in the fisheries of the Algarve region in Portugal and Galicia in Spain. However, landings have declined in recent years, especially in Galicia, where environmental changes in estuaries (Rias) and anthropogenic factors, such as overexploitation, ineffective control of rules and regulations, or illegal fishing practices, have been identified as key contributors to this trend^3^.

In the Iberian Peninsula, the common octopus stands out as one of the most significant fishing resources, enrolling thousands of people on its fisheries along the coastal waters of Galicia (Spain) and mainland Portugal (especially on the Algarve)^4–6^. The common octopus’ fishery is not only a cornerstone of the small-scale fishing sector but also a critical driver of regional economies. In Santa Luzia (Algarve, Portugal), octopus accounts for 99% of both the total weight and value of annual landings^1^. In Galicia, the common octopus fisheries support local economies and small-scale fishing communities, providing substantial economic benefits. Galicia serves as a major hub for octopus exports, supplying both domestic and international demand^7^. The high market value of octopus has led to increased interest in sustainable fishing practices and mariculture to ensure supply stability^8,9^. Efforts to improve sustainability within the octopus’ fishery have contributed to price stabilization and expand access to premium markets^9^.

*Octopus vulgaris* has a short lifespan of 12–18 months with non-overlapping generations, making its populations highly sensitive to environmental variability^10^. In Southern European countries, the management of octopus fisheries primarily falls under the jurisdiction of national and/or local governments, likely reflecting the historical significance of exploitation and the economic importance of this resource^1^. Nevertheless, there is a longstanding acknowledgment that the common octopus has increasingly become a depleted resource, marked by declining catches in the most important supplier countries (Morocco and Mauritania in Northern Africa), largely attributable to overfishing practices^10,11^, illegal fishing practices, misreporting, and unauthorized sales^1^.

*Octopus vulgaris* is a highly versatile feeder and a generalist predator, whose diet is dependent on available prey. The diet of the common octopus encompasses a broad spectrum of species, including bivalves, crustaceans, finfish, and polychaetes^12–14^, while cannibalism and autophagy are also frequently documented in this species^13^. Factors such as seasonal and geographical differences also shape the feeding habits of the common octopus^15^. This feeding plasticity enables the common octopus to rapidly adapt to diverse environments, a trait likely mirrored in its lipidome, making it a potential tool for determining geographic origin.

Globalization of seafood markets allowed a better distribution of products around the world, with once local specific products currently being available in a global market^16^. However, geographic origin is often linked to the quality of specific products in the eyes of consumers, impacting the valuation of these products^17–20^. Additionally, a greater consumer awareness on environmental issues has led to an increased demand for more information regarding harvest or production methods (wild vs. farmed), as well as the management of wild stocks^21,22^. A system providing information at these levels, needs reliable traceability tools to obtain and confirm origin certification of seafood products.

The lipid diversity identified in marine organisms plays a crucial role in several physiological processes, including reproduction, growth, immunological responses, and energy reserves^23^. Lipidome profiles exhibit species-specific characteristics, making their analysis a valuable tool for detecting mislabelling and species substitution in the supply chain, particularly within marine organisms^24,25^. Due to the influence of the surrounding environment in the growth of marine species and the feeding resources they use, unique lipid signatures can also be associated with specific geographic origins^26–28^. Fatty acid and lipid analysis have been successfully used to discriminate the geographic origin of several seafood products, such as the green seaweeds *Ulva* spp.^26^ and *Codium tomentosum*^28^, the brown macroalga *Saccharina latissima*^27^, the common cockle *Cerastoderma edule*^29^, the common octopus *O. vulgaris*^30^, the halophyte plant *Salicornia ramosissima*^31^, or the goose neck barnacle *Pollicipes pollicipes*^19^.

The present study aimed to evaluate the potential use of lipidomic fingerprints as a biochemical tool to pinpoint the geographic origin of common octopus captured and landed in three locations of the Iberian Atlantic coast: Ria de Arousa (RAr), Peniche (Pe), and Santa Luzia (SL). We hypothesized that the polar lipid composition of *O. vulgaris* arm muscle will be unique over different landing locations along the Iberian Atlantic coast and that these natural barcodes could be successfully used to verify claims on its geographic origin.

## Results

### Lipid and phospholipid content

Total lipid content of *O. vulgaris* revealed that Pe (45.86 ± 7.47 mg g^−1^ DW) was the landing location that presented specimens yielding the highest lipid content and in decreasing order RAr and SL (41.53 ± 4.29 mg g^−1^ DW and 40.40 ± 7.53 mg g^−1^ DW, respectively, Table 1). However, no significant differences were found in the lipid content of common octopus’ samples originating from the different locations being studied. Phospholipid quantification expressed as percentage of total lipids, showed significant differences between Pe and the other sites, with Pe recording the lowest value at 36.98% ± 4.59 (Table 1).

**Table 1:** Total lipid and phospholipid content in the arm muscle of *Octopus vulgaris* captured and landed in three different locations along the Iberian Atlantic coast

Sampling sites	Lipid	Phospholipids
	(mg g^−1^ DW)	(% of total lipids)
Ria de Arousa	41.53 ± 4.29	50.61 ± 10.79^a^
Peniche	45.86 ± 7.47	36.98 ± 4.59^b^
Santa Luzia*	40.40 ± 7.53	53.46 ± 5.34^a^

Different letters in phospholipid content indicate significant differences between sampling locations (Tukey’s HSD post hoc test, *p* < 0.05).

^*^Santa Luzia data were previously published in Gaspar et al.^39^

### Polar lipidome profiling

**Fig. 1:**
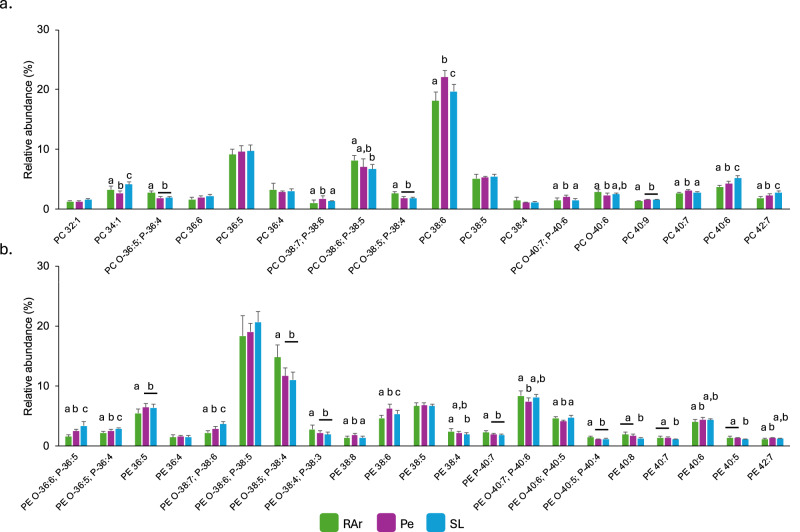
Phosphatidylcholine and phosphatidylethanolamine profile. Relative abundance of phospholipid species of **a** phosphatidylcholine (PC) and **b** phosphatidylethanolamine (PE) identified in common octopus’ (*Octopus vulgaris*) arm muscle captured in three locations along the Iberian Atlantic coast (Ria Arousa—RAr, Peniche—Pe, and Santa Luzia—SL). Data represent relative abundance within the lipid class. Only lipid species with relative abundance >1% were represented. Total lipid species and their relative abundance are summarized in Supplementary Table S1. Different letters indicate significant differences between sampling locations (Tukey’s HSD post hoc test, *p* < 0.05).

**Fig. 2:**
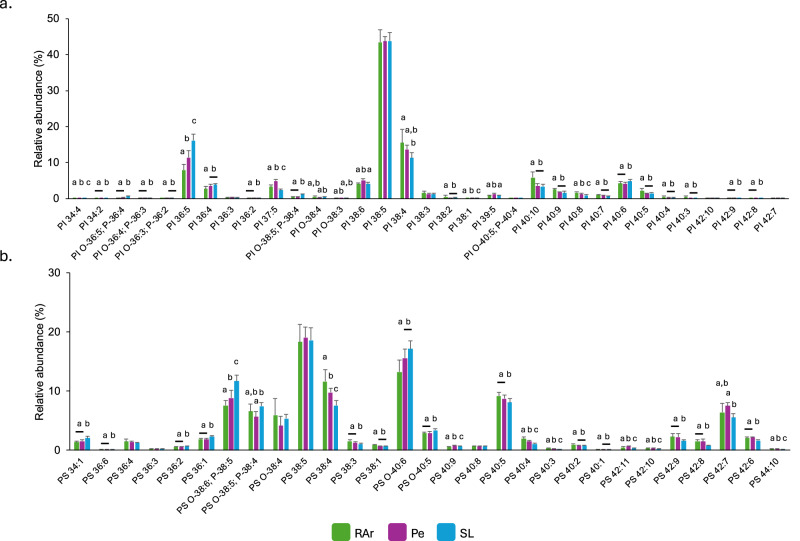
Phosphatidylinositol and phosphatidylserine profile. Relative abundance of phospholipid species of **a** phosphatidylinositol (PI) and **b** phosphatidylserine (PS) identified in common octopus’ (*Octopus vulgaris*) arm muscle captured in three locations along the Iberian Atlantic coast (Ria Arousa—RAr, Peniche—Pe, and Santa Luzia—SL). Data represent relative abundance within the lipid class. Different letters indicate significant differences between sampling locations (Tukey’s HSD post hoc test, *p* < 0.05).

**Fig. 3:**
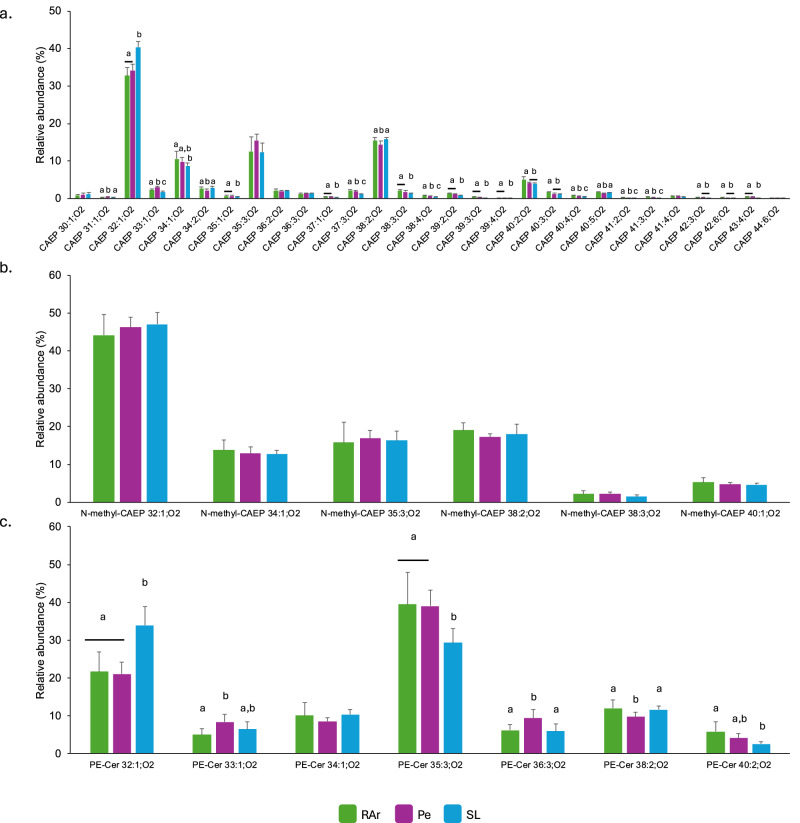
Sphingolipid profile. Relative abundance of lipid species of **a** ceramide aminoethylphosphonate (CAEP), **b** N-methyl ceramide aminoethylphosphonate (*N*-methyl CAEP), and **c** ceramide phosphoethanolamine (PE-Cer) identified in common octopus’ (*Octopus vulgaris*) arm muscle captured in three locations along the Iberian Atlantic coast (Ria Arousa—RAr, Peniche—Pe, and Santa Luzia—SL). Data represent relative abundance within the lipid class. Different letters indicate significant differences between sampling locations (Tukey’s HSD post hoc test, *p* < 0.05).

The analysis performed using HILIC-LC-MS allowed the identification of 372 lipid species (*m/z* values) from 13 polar lipid classes in the lipidome of common octopus’ arm muscle, including glycerophospholipids (Supplementary Fig. S1) and sphingolipids (Supplementary Fig. S2). In the glycerophospholipid category were identified phosphatidylcholine—PC (Fig. 1a) whose main lipid species were PC 38:6 (PC 16:0_22:6) and PC 36:5 (PC 16:0_20:5), lyso PC–LPC (Supplementary Fig. S3a) with LPC 22:6 and LPC 20:5 as the most abundant lipid species; phosphatidylethanolamine—PE (Fig. 1b) with the ether species PE O-38:6/P-38:5 (PE O-18:1/20:5 and PE P-18:0/20:5) and PE O-38:5/P-38:4 (PE O-18:1/20:4 and P-18:0/20:4) showing the highest abundance, lyso PE—LPE with the most abundant species corresponding to LPE O-18:1/P-18:0 and LPE 18:0 (Supplementary Fig. S3b); phosphatidylinositol—PI, with PI 38:5 (PI 18:0_20:5) being the most abundant molecular species in specimens sampled from the three locations surveyed, while the second most abundant in RAr and Pe was PI 38:4 (PI 18:0_20:4) and 36:5 (PI 16:0_20:5) in SL (Fig. 2a); phosphatidylserine—PS with the most abundant lipid species corresponding to PS 38:5 (PS 18:0_20:5) and PS O-40:6 (PS O-18:0/22:6) (Fig. 2b); and phosphatidylglycerol—PG with PG 34:1 (PG 16:0_18:1) as the most abundant lipid species (Supplementary Fig. S4a). In the sphingolipid category (Supplementary Fig. S2) were identified sphingomyelin—SM featuring SM 32:1;O2 (SM 16:1;O2/16:0) and SM 38:2;O2 (SM 16:1;O2/22:1) (Supplementary Fig. S5a); ceramide—Cer with Cer 32:1;O2 (Cer 16:1;O2/16:0) and Cer 30:1;O2 (Cer 18:1;O2/12:0) being the most abundant species (Supplementary Fig. S5b); hexosylceramide—HexCer with HexCer 38:2;O2 (HexCer 16:1;O2/22:1) and HexCer d40:1 being the only species identified in this class (Supplementary Fig. S4b); ceramides aminoethylphosphonate—CAEP which presented CAEP 32:1;O2 (CAEP 16:1;O2/16:0) as the most abundant lipid species in all sampling sites, while the second most abundance was CAEP 38:2;O2 (CAEP 16:1;O2/22:1) in RAr and SL, and CAEP 35:3;O2 (CAEP 19:3;O2/16:0) in Pe (Fig. 3a); *N*-methyl ceramide aminoethylphosphonate—*N*-methyl-CAEP with the lipid species N-methyl-CAEP 32:1;O2 (N-methyl-CAEP 16:1;O2/16:0) and N-methyl-CAEP 38:2;O2 (N-methyl-CAEP 16:1;O2/22:1) showing the highest abundance (Fig. 3b); and ceramide phosphoethanolamine – PE-Cer showing PE-Cer 35:3;O2 (PE-Cer 19:3;O2/16:0) and PE-Cer 32:1;O2 (PE-Cer 16:1;O2/16:0) as the most abundant lipid species (Fig. 3c)] (Supplementary Tables S1 and S2). The ether-linked phospholipids plasmanyl and plasmenyl are isomers with the same *m/z* value, and in this study, they were counted as a single molecular lipid species. They were only distinguished when MS/MS data provided clear evidence to identify one of the isomers.

**Table 2:** Most abundant lipid species within each lipid class, categorized as glycerophospholipids and sphingolipids, identified in the arm muscle of *Octopus vulgaris* collected from three distinct landing sites along the Iberian Atlantic coast: Ria de Arousa (RAr), Peniche (Pe), and Santa Luzia (SL). When the most abundant lipid species differed among locations, the corresponding site is indicated in parentheses

Lipid category		Most abundant lipid species	
Glycerophospholipids	Number of lipid species identified	Lipid species	Molecular lipid species
PC	101	PC 38:6	PC 16:0_22:6
LPC	27	LPC 22:6	LPC 22:6
PE	80	PE O-38:6/P-38:5	PE O-18:1/20:5; PE P-18:0/20:5
LPE	25	LPE O-18:1/P-18:0 (RAr) LPE 18:0 (Pe, SL)	LPE O-18:1; LPE P-18:0 (RAr) LPE 18:0 (Pe, SL)
PI	33	PI 38:5	PI 18:0_20:5
PS	29	PS 38:5	PS 18:0_20:5
PG	4	PG 34:1	PG 16:0_18:1
**Sphingolipids**			
SM	14	SM 32:1;O2	SM 16:1;O2/16:0
Cer	15	Cer 30:1;O2 (RAr) Cer 32:1;O2 (Pe, SL)	Cer 18:1;O2/12:0 (RAr) Cer 16:1;O2/16:0 (Pe, SL)
HexCer	2	HexCer 38:2;O2	HexCer 16:1;O2/22:1
CAEP	29	CAEP 32:1;O2	CAEP 16:1;O2/16:0
N-methyl-CAEP	6	N-methyl-CAEP 32:1;O2	N-methyl-CAEP 16:1;O2/16:0
PE-Cer	7	PE-Cer 35:3;O2 (RAr, Pe) PE-Cer 32:1;O2 (SL)	PE-Cer 19:3;O2/16:0 (RAr, Pe) PE-Cer 16:1;O2/16:0 (SL)

**Fig. 4:**
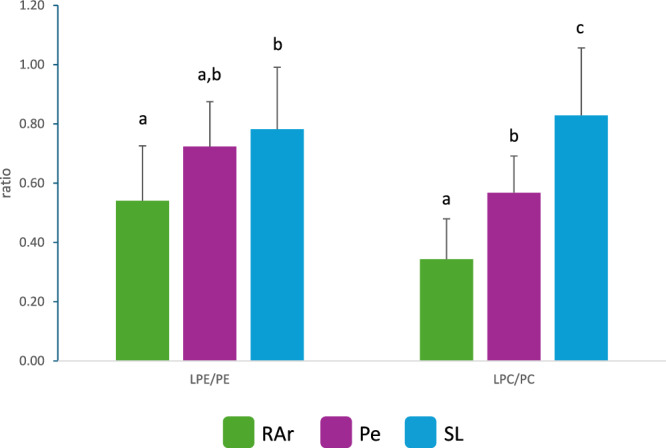
Phospholipid ratios. LPE/PE and LPC/PC ratio identified in lipid extracts of common octopus’ (*Octopus vulgaris*) arm muscle captured in three locations along the Iberian Atlantic coast (Ria Arousa—RAr, Peniche—Pe, and Santa Luzia—SL). Different letters indicate significant differences between sampling locations (Tukey’s HSD post hoc test, *p* < 0.05).

Samples from RAr showed the lowest values of LPE/PE and LPC/PC ratios (0.54 ± 0.19 and 0.34 ± 0.14, respectively), while octopus from SL showed the highest ratio (LPE/PE: 0.78 ± 0.21, LPC/PC: 0.83 ± 0.23). The values of LPE/PE and LPC/PC ratios for Peniche samples were 0.72 ± 0.15 and 0.57 ± 0.12, respectively (Fig. 4).

**Fig. 5:**
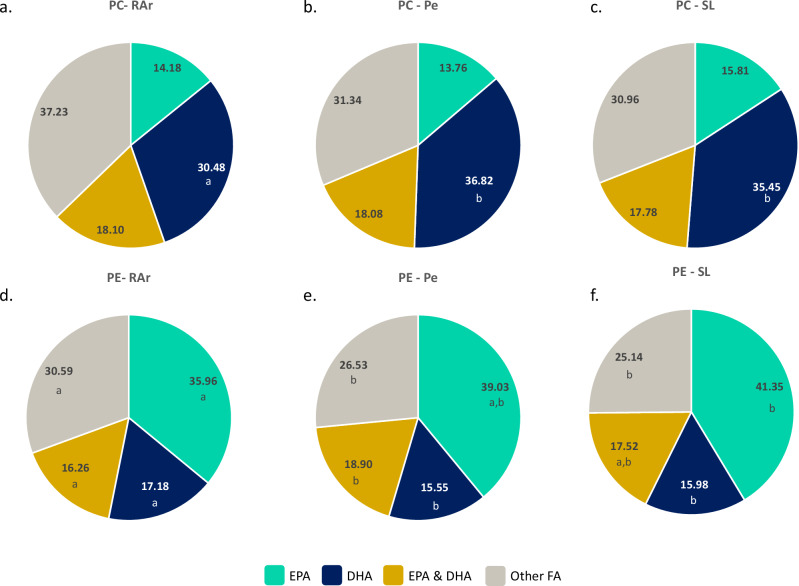
Distribution of EPA and DHA in phosphatidylcholine and phosphatidylethanolamine lipid species. Relative abundance (%) of lipid species containing EPA (20:5 n-3), DHA (22:6 n-3), both (EPA & DHA) or other fatty acids (FA) in the fatty acyl composition of phosphatidylcholine (PC) in samples of common octopus’ (*Octopus vulgaris*) arm muscle from **a** Ria Arousa (RAr), **b** Peniche (Pe), **c** Santa Luzia (SL) and in the composition of phosphatidylethanolamine (PE) in samples from **d** RAr, **e** Pe, **f** SL. Lipid species whose fatty acyl composition was not identified were included in other FA. Different letters under relative abundance indicate significant differences between sampling locations (Tukey’s HSD post hoc test, *p* < 0.05).

**Fig. 6:**
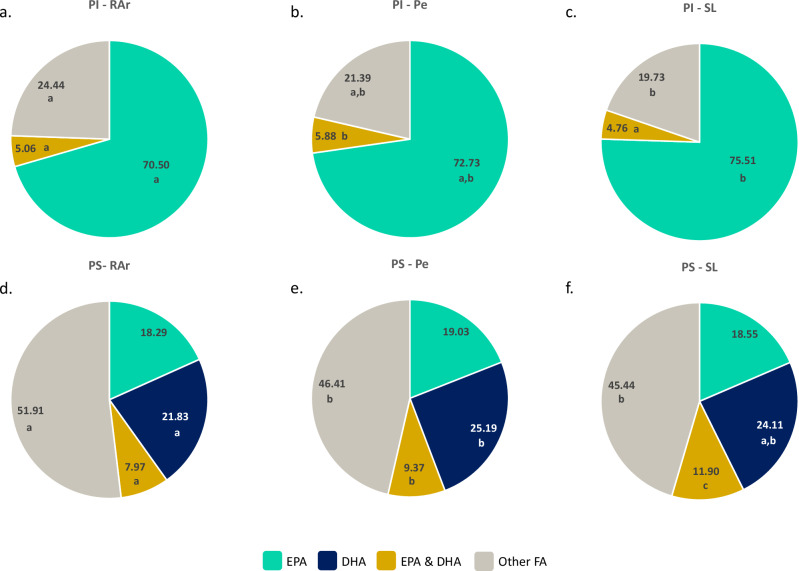
Distribution of EPA and DHA in phosphatidylinositol and phosphatidylserine lipid species. Relative abundance (%) of lipid species containing EPA (20:5 n-3), DHA (22:6 n-3), both (EPA & DHA) or other fatty acids (FA) in the fatty acyl composition of phosphatidylinositol (PI) in samples of common octopus’ (*Octopus vulgaris*) arm muscle from **a** Ria Arousa (RAr), **b** Peniche (Pe), **c** Santa Luzia (SL) and in the composition of phosphatidylserine (PS) in samples from **d** RAr, **e** Pe, **f** SL. Lipid species whose fatty acyl composition was not identified were included in other FA. Different letters under relative abundance indicate significant differences between sampling locations (Tukey’s HSD post hoc test, *p* < 0.05).

The relative abundance of essential fatty acids, namely eicosapentaenoic acid (EPA, 20:5 *n*-3) and docosahexaenoic acid (DHA, 22:6 *n*-3) in the fatty acyl composition of the main phospholipid classes was determined. PC was the phospholipid class with the highest percentage of lipid species containing DHA in their composition (31% in RAr, 37% in Pe and 36% in SL, Fig. 5a, b, c), while, in PE the lipid species containing EPA in their structure accounted for 36% in RAr, 39% in Pe and 41% in SL (Fig. 5d, e, f). Most of the lipid species in PI presented EPA as an esterified fatty acid in their structure (70% in RAr, 73% in Pe, and 76% in SL, Fig. 6a, b, c). No PI lipid species were identified containing only DHA (Fig. 6a, b, c). In PS, the relative abundance of lipid species containing EPA (18% in RAr, 19% in Pe, 16% in SL) was slightly lower than those containing DHA (22% in RAr, 25% in Pe, 24% in SL, Fig. 6d, e, f).

**Fig. 7:**
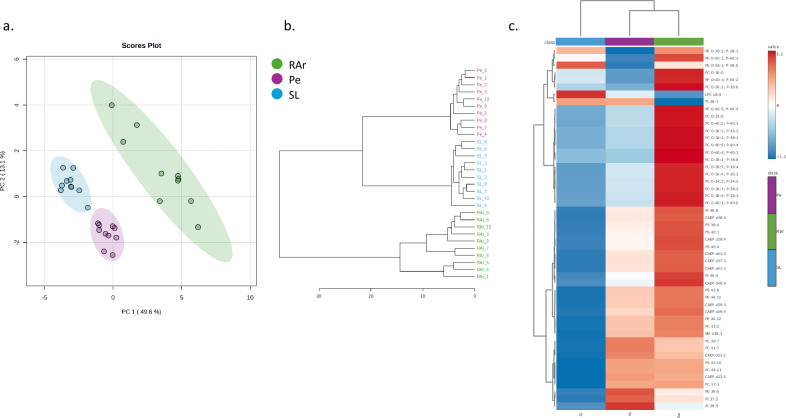
Multivariate analysis of the polar lipidome of *Octopus vulgaris.* **a** Principal component analyses (PCA), **b** dendrogram, and **c** hierarchical clustering heatmap of the top 50 lipid species sorted by analysis of variance test displaying the lowest *p*-values of log-transformed normalized extracted-ion chromatogram (XIC) areas of total polar lipid species identified in the arm muscle of *Octopus vulgaris* samples captured in three locations along the Iberian Atlantic coast (Ria Arousa—RAr, Peniche—Pe, and Santa Luzia—SL).

The ANOVA test of normalized extracted ion chromatograms (XIC) areas of polar lipid revealed the existence of significant differences between locations in 268 of the 372 identified lipid species (Supplementary Table S2). The eigenvalues of the two principal components of the principal component analysis (PCA) analysis performed using the normalized XIC areas of polar lipid species explained 62.7% of total variance, with principal component 1 (PC1) and principal component 2 (PC2) axes explaining 49.6% and 13.1%, respectively (Fig. 7a). PC1 allowed the separation of RAr samples from those originating from Pe and SL, while PC2 separated Pe from SL samples. Samples originating from RAr displayed a higher dispersion in the PCA plot when compared with the other two locations (Fig. 7a). These results were supported by the hierarchical clustering dendrogram (Fig. 7b) and heatmap (Fig. 7c) performed using the top 50 lipid species sorted by analysis of variance test displaying the lowest *p*-values (Supplementary Table S2). The lipid species that most contributed for the discrimination of the samples and their clustering were ether-linked phospholipids, of PC and PE, diacyl forms of PS and PI and the sphingolipid CAEP. All of them were more abundant in samples from RAr, except the lipid species LPC 18:4, PI 38:1, PI39:5, and PE O-38:1/P-38:0, which were more expressed in the samples from Pe and SL (Fig. 7c).

## Discussion

The lipidome of marine organisms is highly influenced by prevailing conditions in their habitat, which are shaped by various factors, such as temperature, diet, feeding behavior, life stage, or physiological state^32,33^. Total lipid content in the arm muscle of common octopus was not influenced by environmental factors across the studied locations, suggesting that lipid levels in arm muscle remain constant under different growth conditions. The relative abundance of phospholipids in total lipid extracts (~50%) from RAr and SL aligns with previous findings reported by Prato et al.^34^ for wild-sourced *O. vulgaris*. In contrast, samples from Pe showed the lowest phospholipid content, resembling levels reported for specimens cultured using a mixed diet of crab, fish, and mussel by Prato et al.^34^. These results suggest that individuals from Pe likely underwent dietary-driven shifts. Phospholipids, as essential components of cell membranes, play a fundamental role in maintaining membrane integrity and function^35^. These polar lipids contribute to membrane permeability, facilitating the exchange of nutrients and ions, and regulate metabolism, physiology, and energy production^36,37^. Environmental changes in marine ecosystems influence polar lipid composition, with shifts in phospholipid profiles serving as biochemical markers of an organism’s physiological state^35^. Stressors, such as salinity fluctuations^38^ and dietary changes^34^, have been linked to variations in phospholipid content.

The polar lipidome of *O. vulgaris* was recently characterized in detail through a comprehensive study by Gaspar et al.^39^. The present study revealed consistent findings across the three sampling locations. The lipidome of *O. vulgaris* presented a high abundance of ether-linked lipids, especially in PC, PE, LPE, and PS. Depending on the groups at *sn*-1 of the glycerol backbone, the ether-linked phospholipids can be divided into two subclasses: 1-O-alkyl-2-acyl (plasmanyl, identified with O in the nomenclature), and 1-O-(alk-1′-enyl)-2-acyl (plasmenyl or plasmalogen, identified with P in the nomenclature). Specifically, plasmalogens contribute to cellular membrane structure-mediated functions and are key biomolecules in the response to oxidative stress, considered important endogenous anti-oxidant molecules, that provide protection to membrane lipids against oxidation^40–42^. These lipids enhance cellular adaptability by preserving membrane integrity under stress conditions^43^. Ether-linked phospholipids exhibited the greatest variation among sampling areas, likely reflecting metabolic adjustments in *O. vulgaris* to different environmental conditions or diets.

In marine organisms, the LPC/PC and LPE/PE ratios highlight the dynamic interplay between lipid metabolism and external factors. These ratios have significant biological implications, as they reflect lipid metabolism, oxidative stress, environmental adaptation, and seafood quality control^47^. Elevated LPC/PC ratios, as it was observed in SL samples, indicate PC hydrolysis, often due to enzymatic activity (e.g., phospholipase A2) or oxidative stress, affecting membrane stability and fluidity^47,48^. Additionally, elevated LPE/PE ratios reflect PE breakdown, which compromises membrane structure and function. This is particularly evident under stress conditions like oxidative damage or improper tissue storage^47,48^. The elevated LPC/PC and LPE/PE ratios in SL samples suggest that octopus from this landing site likely experienced stress conditions, such as food shortage, temperature fluctuations, habitat changes, or even improper storage after death.

The main phospholipid classes, namely PC and PE, showed DHA and EPA in their most abundant lipid species. These essential polyunsaturated fatty acids (PUFA) were predominant in both wild and reared octopuses^34,44^. *Octopus vulgaris* accumulates DHA and EPA primarily through its diet of crustaceans, molluscs, and fish. However, its predatory nature results in higher DHA content compared to EPA^45^. The high concentration of these fatty acids in *O. vulgaris* tissues plays a crucial role in nervous system development and function^46^.

The lipid species that contributed the most for the discrimination recorded between sampling sites were mainly ether-linked phospholipids (i.e., PC and PE), suggesting an adaptation of cell membranes to the different sampling environments. The high content of ether-linked PC in RAr, when compared to other locations, can be related to the higher availability of bivalves, as these are farmed using aquaculture systems in this area. Marine invertebrates contain a high diversity of ether-linked phospholipids in their lipidomes^42,49^, which are especially abundant in molluscs^50,51^. Plasmalogens were estimated to represent 33%, 54% and 63% of total PC, PE, and PS in *Mytilus edulis*, respectively^51^. Different capture locations often showcase contrasting oceanographic dynamics and available preys, which can ultimately shape the lipidome of organisms that inhabit them. The Galician coast, located in the northwest part of Spain, is characterized by the Rías (drowned river valleys), which create a unique coastal landscape and provide rich habitats for marine life and favor aquaculture practices targeting the grow-out of bivalves. For decades, the common octopus has been harvested along the Galician coastline, with ports in the Rías Baixas, such as RAr, traditionally experiencing high landings^5^. These environments are highly influenced by upwelling events, ranking RAr as a highly productive marine ecosystem^52,53^. It is worth highlighting that RAr is the largest ria in the coast of Galicia and features an impressive aquaculture production of Mediterranean mussel *Mytilus galloprovincialis*, accounting for nearly 70% of all mussel rafts in Galicia^54^. Mussel aquaculture significantly influences the diet of common octopus in these locations, as mussel production rafts can attract possible octopus’ prey^55^, therefore enhancing feeding opportunities. The influence of raft culture in the diet of other invertebrates (e.g., the harbor crab *Liocarcinus depurator*) has already been reported^56^. Therefore, samples from RAr presenting a higher abundance of ether-linked PC (plasmanyl and plasmenyl) may be associated with the octopus’ diet in this area.

The CAEP molecular species that contributed to discriminate between landing areas were particularly abundant in samples originating from RAr. This sphingolipid class is not very common in marine organisms, although it has been identified in some mollusc, echinoderm, and cnidarian species^57^. The most discriminating CAEP lipid species included the sphingoid bases 16:1;O2 and 19:3;O2 in their composition, which are the most frequently found in marine invertebrates^58^. CAEP are very abundant in different molluscs, such as the squid *Uroteuthis chinensis*^58^ and the Mediterranean mussel *M. galloprovincialis*^59^. These molecules act as protective cell membrane agents due to their endurance against hydrolytic enzymes, thus enabling cellular adaptation to environmental changes^60^. CAEP and sterols have been suggested as relevant molecules to form lipid rafts in the cell membranes of marine invertebrates^49^, contributing to the regulation of their fluidity. In nudibranch molluscs, PI and CAEP have been identified as a conservative part of their polar lipidome, without experiencing any major influence by diet^61^. As such, the differences observed in the CAEP composition between samples originating from different geographic areas may most likely be related to environmental changes that have promoted pronounced shifts in the cell membranes of *O. vulgaris*.

In conclusion, the present study revealed the polar lipidome profile of *O. vulgaris* and its compositional variations across three landing sites along the Iberian Atlantic coast. While total lipid content in *O. vulgaris* arm muscle remained consistent across locations, phospholipid levels were lower in specimens landed in Pe, most likely due to environmental or dietary differences. Additionally, octopus from SL exhibited the highest LPE/PE and LPC/PC ratios, suggesting an exposure to stressors or inadequate storage conditions. The dominant polar lipid species were rich in essential fatty acids, such as EPA and DHA, highlighting their potential as valuable sources of these PUFA. This study represents a first step in evaluating lipidomics as a reliable tool for the discrimination of the geographic origin of *O. vulgaris*, as the composition of its lipidome reflects environmental conditions and dietary resources in different fishing grounds. Key membrane lipid classes in marine invertebrates, including PC, PE, CAEP, and ether-linked phospholipids, significantly contributed to the differentiation among sampling locations. Although the findings of this study highlight the potential of lipidomic profiling of *O. vulgaris* arm muscle to indicate geographic origin, further validation is needed to evaluate its effectiveness, compliance with traceability standards, and feasibility for routine application along the seafood supply chain. Expanding the analysis to include specimens from the Mediterranean and Moroccan/Mauritanian coasts, along with seasonal and long-term sampling, would provide deeper insights into how environmental and trophic factors influence the lipidome and biochemical phenotypes of *O. vulgaris*.

## Methods

### Sampling

**Fig. 8:**
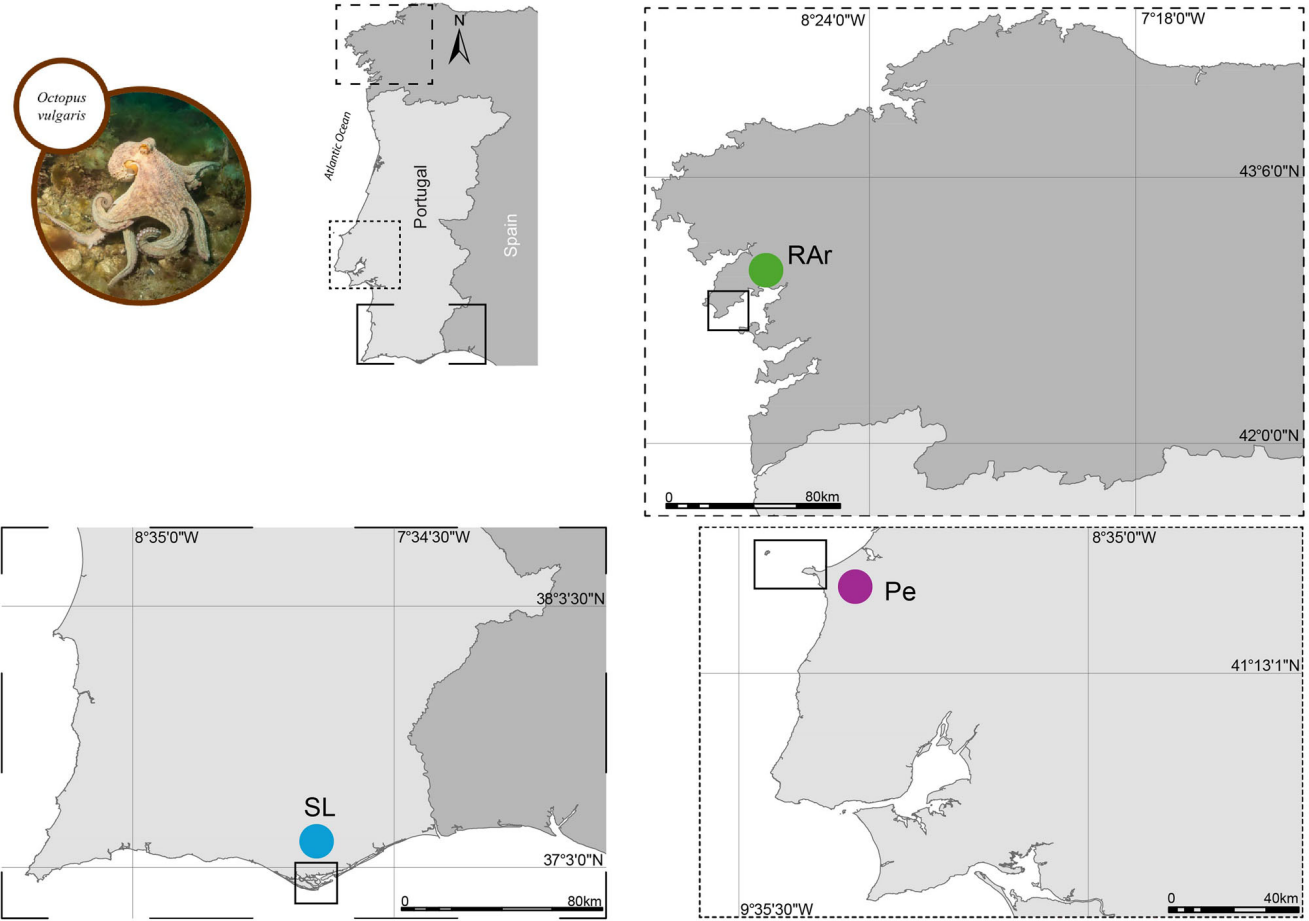
Landing locations. Sampling areas of *Octopus vulgaris* specimens along the Atlantic coast of the Iberian Peninsula: Ria de Arousa, Peniche, and Santa Luzia.

Samples of *O. vulgaris* were sourced from fisher’s landing operating on three different areas along the Atlantic coast of Galicia (Spain) [Ria de Arousa (RAr, Porto de Ribeira)] and Portugal [Peniche (Pe) and Santa Luzia (SL)] (Fig. 8), in the summer of 2018. A total of 3 locations and 10 individuals of common octopus (*n* = 10) per location were sampled (3 × 10 = 30). Sampled specimens were transported in coolers to the laboratory immediately after being landed in the different fishing harbors. The fourth right arm counted from the sagittal plane, front to back, was cut and stored at –80 °C. Samples were subsequently freeze-dried (CoolSafe 55–9 L Pro, Labogene, Lillerød, Denmark) and stored at –80 °C for further analysis.

All octopuses were collected, slaughtered, and maintained refrigerated by duly licensed professional fishers while still onboard, being already dead when landed in the fishing harbor; as such, ethical issues concerning animal experimentation and welfare do not apply to the present study.

### Lipid extraction

Freeze-dried samples of *O. vulgaris* arm muscle were individually homogenized using a mortar grinder (RM 200, Retsch, Hann, Germany). Between the homogenization of samples, the mortar grinder was carefully cleaned with silicate followed by alcohol (70%) to avoid cross-contamination. A modified Bligh and Dyer^62^ method was used to extract total lipids^39^. An amount of 50 mg of *O. vulgaris* arm muscle was mixed with 2.5 mL of methanol (MeOH) and 1.25 mL of dichloromethane (CH_2_Cl_2_) in a glass centrifuge tube. After homogenization and sonication for 1 min, using an automatic ultrasonic frequency of 35 kHz (Bandelin, Sonorex, RK 100, Berlin, Germany), the sample was incubated on ice on a rocking platform shaker for 30 min. The tube was then centrifuged at 568 × *g* for 10 min at room temperature (UNIVERSAL 320 R, Hettich, Tuttlingen, Germany). The organic phase containing the lipids was collected in a new tube and the biomass residue was re-extracted. A volume of 1.25 mL of CH_2_Cl_2_ and 1.25 mL of Mili Q water was added to the organic phases containing the lipid. After homogenization and centrifugation for 10 min at 568 × *g* to promote phase separation, the organic phases containing the lipids were collected in a new tube and the aqueous phase of the first extraction was re-extracted with 1.88 mL of CH_2_Cl_2_ followed by homogenization and centrifugation for 10 min 568 × *g* to recover the organic phase. The total lipid extract was dried using a speed vacuum (UNIVAPO-100H coupled with UNIJET II refrigerated aspirator) and transferred to previously dried and weighed dark vials. The lipid extracts were stored at –20 °C. The total lipid content was estimated by gravimetry and used to determine the lipid content per dry weight of biomass (µg mg^−1^ DW).

### Phospholipid quantification

The content of phospholipids in total lipid extracts was performed through the phosphorus assay^63^. Total lipid extract was resuspended in 300 µL of CH_2_Cl_2_ and a volume of 10 µL of lipid extract was transferred to a glass tube and dried under a nitrogen stream. Then, 125 µL of 70% perchloric acid was added followed by incubation at 180 °C for 60 min in a heating block. After cooling at room temperature, 825 µL of MiliQ water, 125 µL of 2.5% aqueous solution of ammonium molybdate ((NH_4_)_6_ Mo_7_O_24_·4H_2_O) and 125 µL of 10% ascorbic acid were added to the glass tubes. The standards were prepared using 0.1–2 µg of phosphate (standard solution of NaH_2_PO_4_·2H_2_O, 100 µg phosphorus mL^−1^) to prepare a calibration curve and followed the same treatment as the samples. Samples and standards were incubated at 100 °C in a water bath (Precisterm, JP Selecta) for 10 min, followed by cooling in cold water. The absorbance of samples and standards was measured at 797 nm using a microplate ultraviolet-visible spectrophotometer (Multiskan GO, Thermo Scientific).

The amount of phospholipids was estimated by multiplying the amount of phosphorus in each sample by 25, the conversion factor between phosphorus and phospholipids^64^.

### Hydrophilic interaction liquid chromatography-mass spectrometry

Lipid extracts were analyzed by hydrophilic interaction liquid chromatography-mass spectrometry (HILIC-LC-MS) on an Ultimate 3000 Dionex ultra-high-performance liquid chromatography (UHPLC) system (Thermo Fisher Scientific) with an autosampler coupled online to a Q-Exactive mass spectrometer with Orbitrap ® technology (Thermo Fisher Scientific). A two-mobile-phase solvent system was used to perform the analysis: a mobile phase A consisting of a mix of water, acetonitrile, and methanol (25/50/25, by volume) and a mobile phase B consisting of acetonitrile and methanol (60/40, by volume), with both phases presenting 5 mM ammonium acetate. To perform the LC-MS analysis an amount of lipid extract corresponding to 5 µg of phospholipids was dissolved in CH_2_Cl_2_ (1 µg μL^−1^) and mix with 75 µL of the starting eluent (95/5, B/A, by volume) and 4 µL of each lipid standard [ceramide (Cer d18:1_17:0), 0.04 µg; 1,2-dimyristoyl-sn-glycero-3-phosphate(dMPA), 0.08 µg; 1,2-dimyristoyl-sn-glycero-3-phospho-(10-rac-)glycerol (dMPG), 0.012 µg; lysophosphatidylcholine (LPC), 0.02 µg; 1,2-dimyristoylsnglycero-3-phosphoethanolamine (dMPE), 0.02 µg) (Avanti Polar Lipids, Inc., Alabaster, AL). The samples were injected into an ACE HILIC-N microbore column (100 × 1.0 × 3 µm) at a flow rate of 50 µL min^−1^ and a temperature of 35 °C. Initially, 5% of mobile phase A was held isocratically for 2 min, followed by a linear increase to 70% of mobile phase A within 11 min and a new linear increase to 90% within 7 min, maintained for 30 min, and returning to the initial conditions in 5 min, being held for an additional 5 min.

The Orbitrap mass spectrometer was operated using positive/negative switching toggles between positive (electrospray voltage 3.0 kV) and negative (electrospray voltage −2.7 kV) ion modes with a capillary temperature of 250 °C and a sheath gas flow of 15 U. In MS experiments, a high resolution of 70,000 was used, as well as an automatic gain control (AGC) target of 1 × 10^6^. In tandem mass spectrometry (MS/MS), a resolution of 17,500 and AGC target of 1 × 10^5^ were used and cycles consisted of one full-scan mass spectrum and 10 data-dependent MS/MS scans were repeated continuously throughout the analysis with the dynamic exclusion of 60 s and an intensity threshold of 2 × 10^4^. Normalized collision energy ^TM^ (CE) ranged between 25, 30, and 35 eV. Data acquisition was performed using the Xcalibur data system (V3.3, Thermo Fisher Scientific, USA). The molecular identification of lipid species was performed by interpretation of the HILIC–ESI–MS/MS spectra, through the typical fragmentation patterns of the polar head group and the fatty acyl chains inherent in the structure of each molecular species^39^, LC-MS expected retention time, and mass accuracy (Qual Browser) with an error of ≤5 ppm.

### Data analysis

Raw data processing was performed using the MZmine 2.53 software^65^. The MS raw data were pre-processed by filtering and smoothing. Peak assignment and ion identification were based on mass accuracy and performed against in-house polar lipid databases. The mass list was filtered, then peaks were detected and processed. The parameters set for MZmine 2.53 were the following: minimum peak height, above 1 × 10^4^; join alignment; allowable error of retention time, 0.5 min; acceptable error of *m/z* (*m/z* tolerance) 5 ppm. Integrated peak area values from lipid species were exported using the comma-separated values (.csv) format. Integrated peak areas of each lipid species were exported and data were normalized by dividing peak areas from extracted ion chromatograms (XIC) for each lipid species by the peak area of selected internal standard for lipid classes as follows: PG, PI with dMPG; CAEP, *N*-methyl-CAEP, PC, PE, PE-Cer, and PS with dMPE; Cer and HexCer with Cer d18:1_17:0; LPC, LPE, and SM with LPC.

### Statistical analysis

One-way ANOVA was performed to assess significant differences in the content of total lipids (mg g^−1^ DW), phospholipids (% of total lipids), relative abundance of lipid species within the corresponding lipid class, and normalized XIC areas of identified lipid species between landing sites. Shapiro–Wilks and Bartlett’s tests were performed to evaluate ANOVA assumptions of normality and homogeneity of variance, respectively.

Normalized XIC areas of polar lipids were processed using Metaboanalyst 6.0^66^. Missing values were replaced by 1/5 of the minimum positive value of each variable. Data filtering was performed to remove variables that evidenced a low repeatability by relative standard deviation (RSD = SD/mean), followed by log transformation. Principal component analysis (PCA) was performed to visualize the general 2D clustering of *O. vulgaris* samples from three different locations. A hierarchical clustering dendrogram and a heatmap were performed using Euclidean distances and the Ward clustering algorithm. Heatmap provided a better understanding of the significance of the variables in separating different landing sites. The top 50 molecular species were ranked using *p*-values from an ANOVA test.

## Supplementary information

Supplementary Information

Supplementary Information

Supplementary Information

## Data Availability

The datasets generated during the current study are available from the corresponding author (felisa.rey@ua.pt) on reasonable request.

## Abbrevaitions

CAEP: Ceramide aminoethylphosphonate
Cer: Ceramide
CH_2_Cl_2_: Dichloromethane
DHA: Docosahexaenoic acid
dMPA: 1,2-dimyristoyl-sn-glycero-3-phosphate
dMPE: 1,2-dimyristoylsnglycero-3-phosphoethanolamine
dMPG: 1,2-dimyristoyl-sn-glycero-3-phospho-(10-rac-glycerol)
EPA: Eicosapentaenoic acid
HexCer: Hexosylceramide
HILIC: Hydrophilic interaction liquid chromatography
LPC: Lyso-phosphatidylcholine
LPE: Lyso-phosphatidylethanolamine
MeOH: Methanol
MS: Mass spectrometry
MS/MS: Tandem mass spectrometry
*N*-methyl-CAEP: *N*-methyl ceramide aminoethylphosphonate
PC: Phosphatidylcholine PCA Principal component analysis
PE: Phosphatidylethanolamine
Pe: Peniche
PE-Cer: Ceramide phosphoethanolamine
PG: Phosphatidylglycerol
PI: Phosphatidylinositol
PS: Phosphatidylserine
PUFA: Polyunsaturated fatty acids
RAr: Ria de Arousa
SL: Santa Luzia
SM: Sphingomyelin
UHPLC: Ultra-high-performance liquid chromatography
XIC: Extracted ion chromatograms
