# Supplementary material for: Lipidomic signatures in Octopus vulgaris arm muscle reveal geographic variation along the Iberian Atlantic Coast
Source: NPJ Sci Food. 2025 Aug 9;9:173. doi: 10.1038/s41538-025-00520-w (PMC12335516; doi:10.1038/s41538-025-00520-w)
Supplement: Supplementary file 1 — Supplementary Information [file 41538_2025_520_MOESM1_ESM.pdf]

Supplementary Figures

Lipidomic signatures in *Octopus vulgaris* arm muscle reveal geographic variation along the Iberian Atlantic Coast

Felisa Rey[1,2*], Luís Gaspar[1], Fernando Ricardo[3], Cristina Pita[4,5], Maria do Rosário Domingues[1,2], Ricardo Calado[3]

1. CESAM - Centre for Environmental and Marine Studies, Department of Chemistry, University of Aveiro, Campus Universitário de Santiago, 3810-193 Aveiro, Portugal
2. Mass Spectrometry Centre & LAQV-REQUIMTE, Department of Chemistry, University of Aveiro, Campus Universitário de Santiago, 3810-193 Aveiro, Portugal
3. ECOMARE - Laboratory for Innovation and Sustainability of Marine Biological Resources, CESAM - Centre for Environmental and Marine Studies, Department of Biology, Campus Universitário de Santiago, University of Aveiro, 3810-193 Aveiro, Portugal
4. CESAM - Centre for Environmental and Marine Studies, Department of Environment and Planning, University of Aveiro, Campus Universitário de Santiago, 3810-193 Aveiro, Portugal
5. Institute of Marine Research (IIM-CSIC), Rúa de Eduardo Cabello, 6, 36208 Vigo, Pontevedra, España

*Felisa Rey: felisa.rey@ua.pt

Glycerophospholipids

a) PC 16:0_22:6

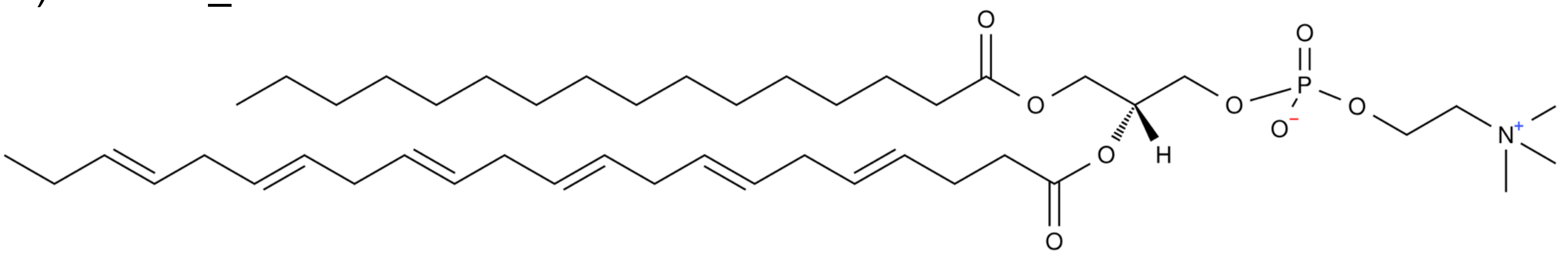

b) LPC 22:6

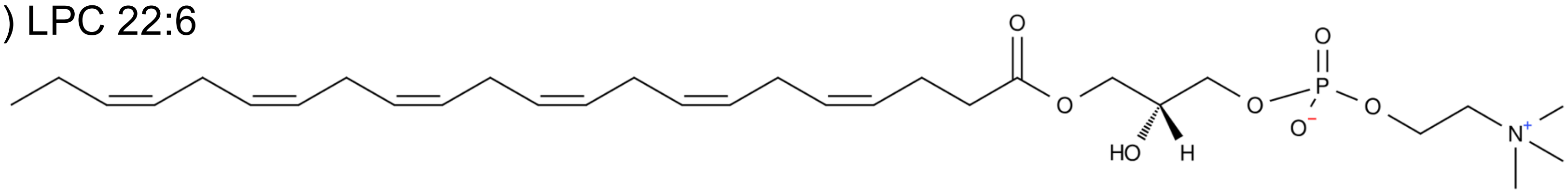

c) PE P-18:0/20:5

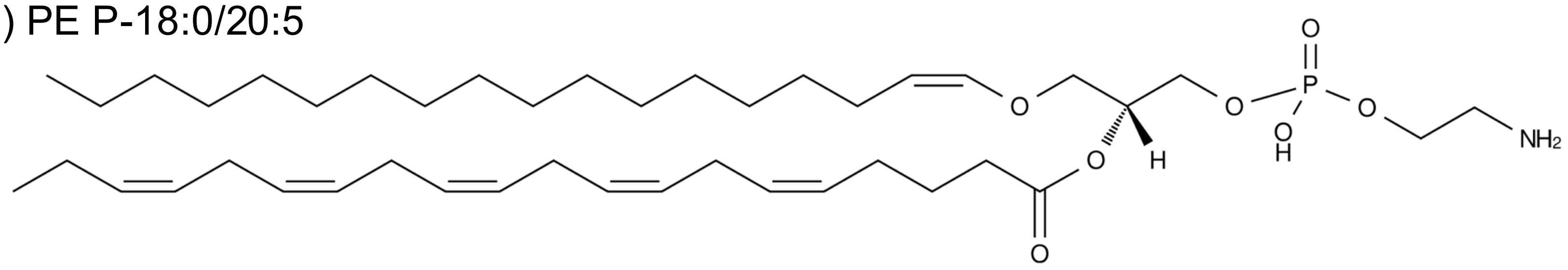

d) LPE O-18:1

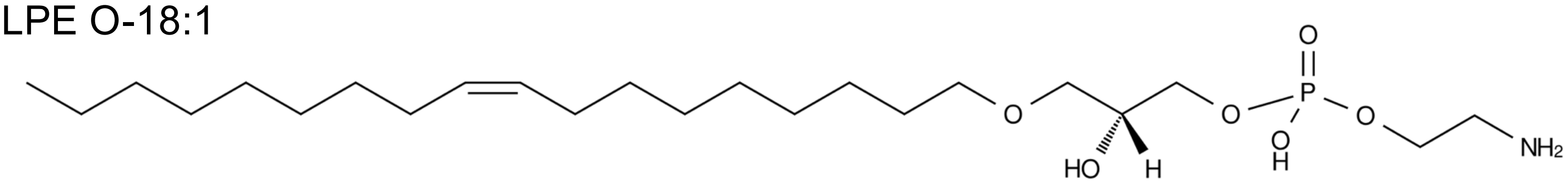

e) PI 18:0_20:5

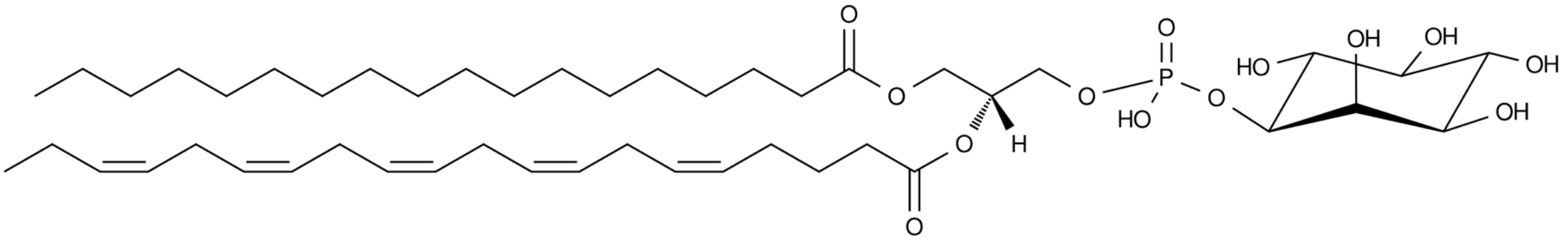

f) PS 18:0_20:5

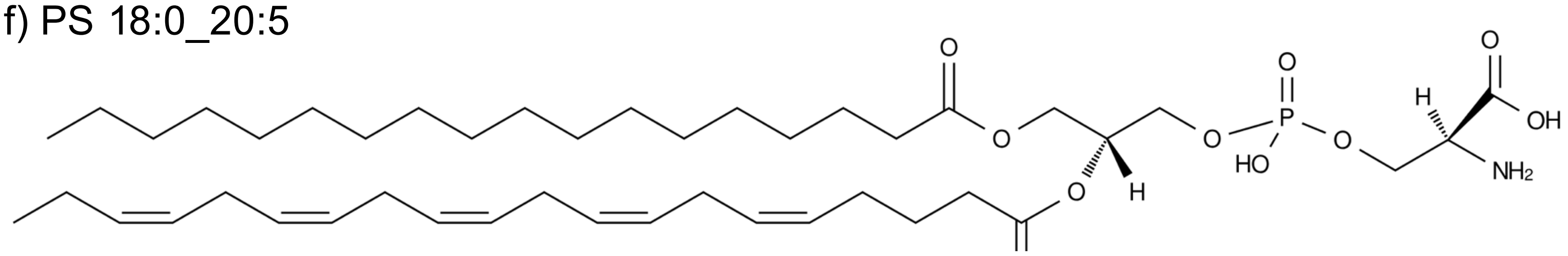

g) PG 16:0_18:1

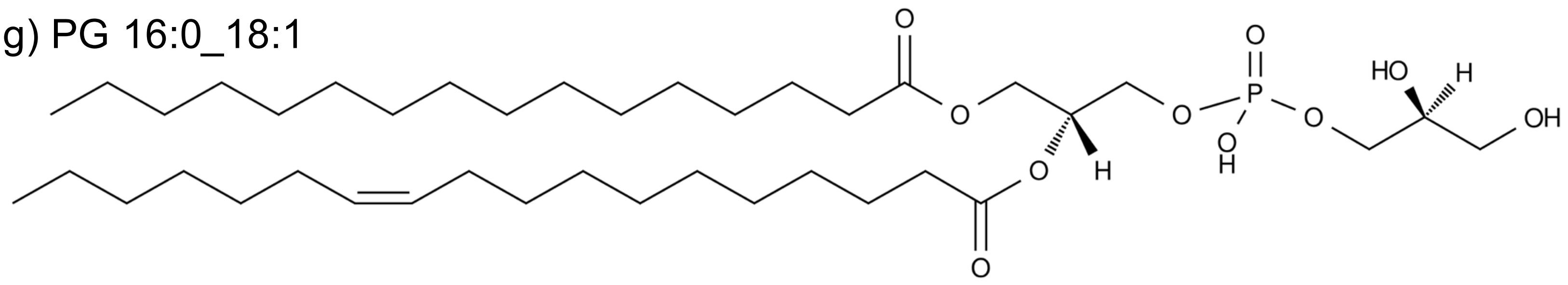

Supplementary Figure S1 Glycerophospholipids indenified in the arm muscle of *Octopus vulgaris. a*) Phosphatidylcholine (PC); b) lyso PC (LPC); c) phosphatidylethanolamine (PE); d) lyso PE (LPE); e) phosphatidylinositol (PI); f) phosphatidylserine (PS) and g) phosphatidylglycerol (PG).

Sphingolipids

a) SM 16:1;O2/16:0

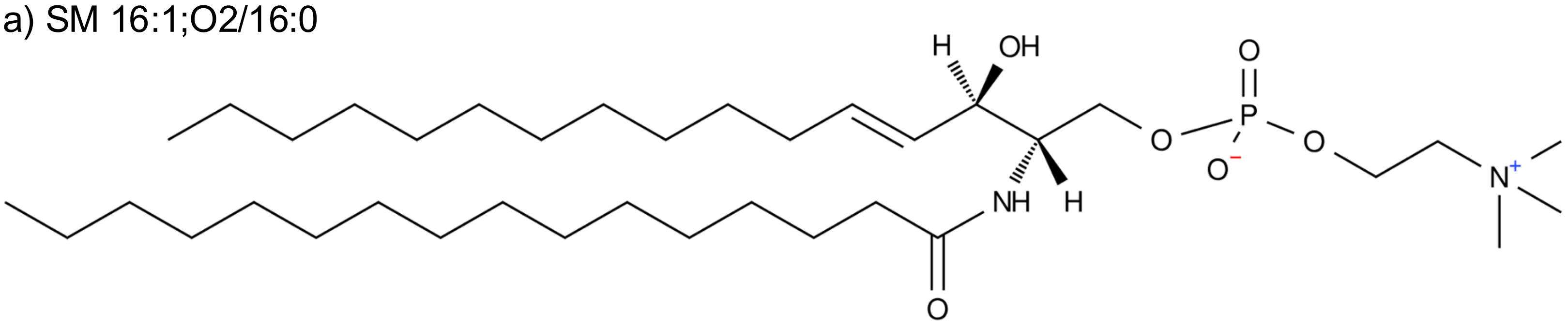

b) Cer 18:1;O2/12:0

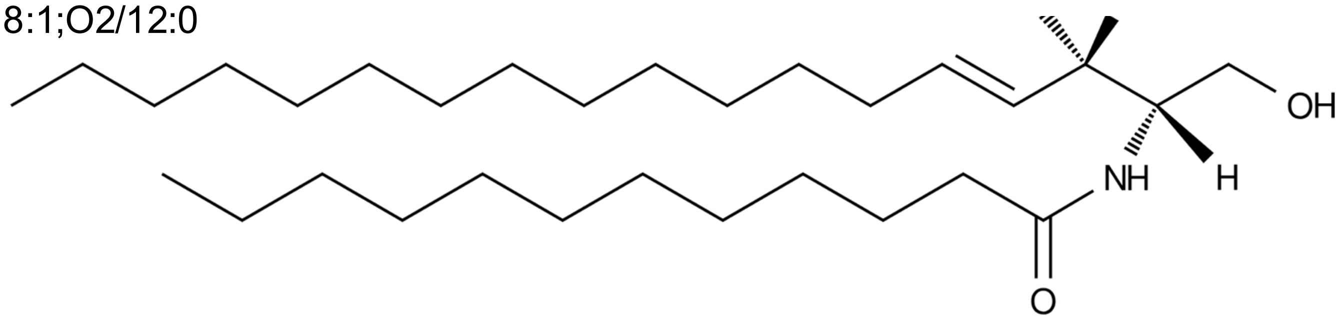

c) HexCer 16:1;O2/22:1

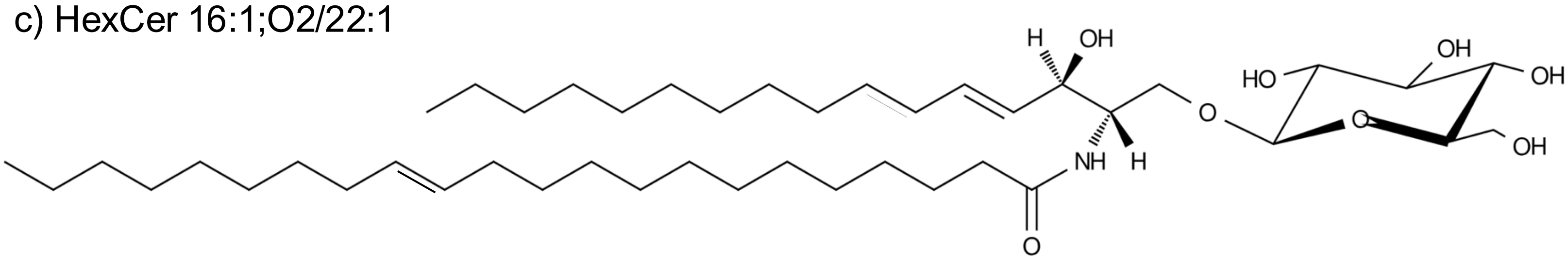

d) CAEP 16:1;O2/16:0

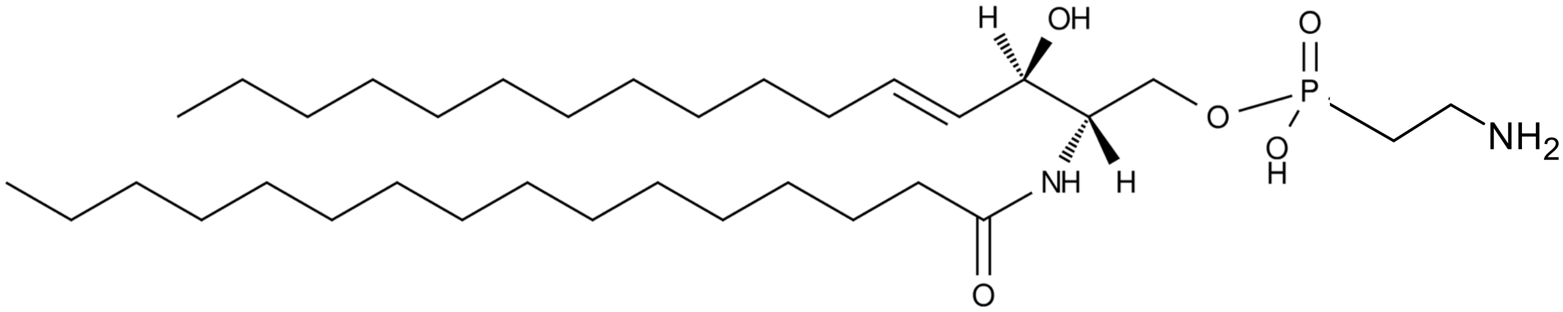

e) N-methyl-CAEP 16:1;O2/16:0

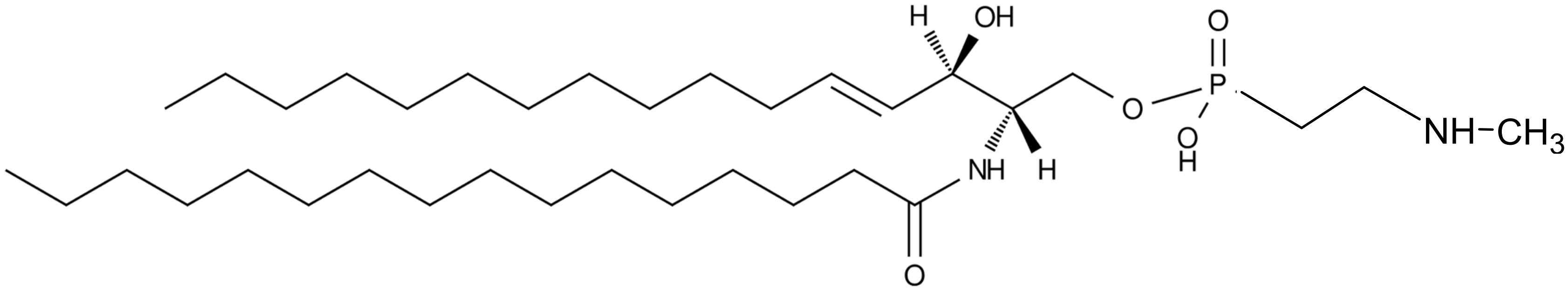

f) PE-Cer 16:1;O2/16:0

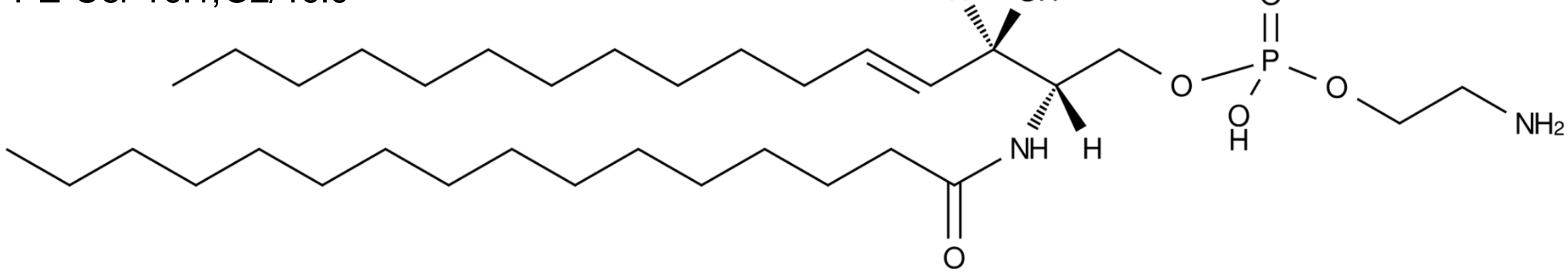

Supplementary Figure S2 Sphingolipids indenified in the arm muscle of *Octopus vulgaris*. *a*) Sphingomyelin (SM); b) ceramide (Cer); c) hexosylceramide (HexCer); d) ceramide aminoethylphosphonate (CAEP); e) *N*-methyl CAEP and f) Ceramide phosphoethanolamine (PE-Cer).

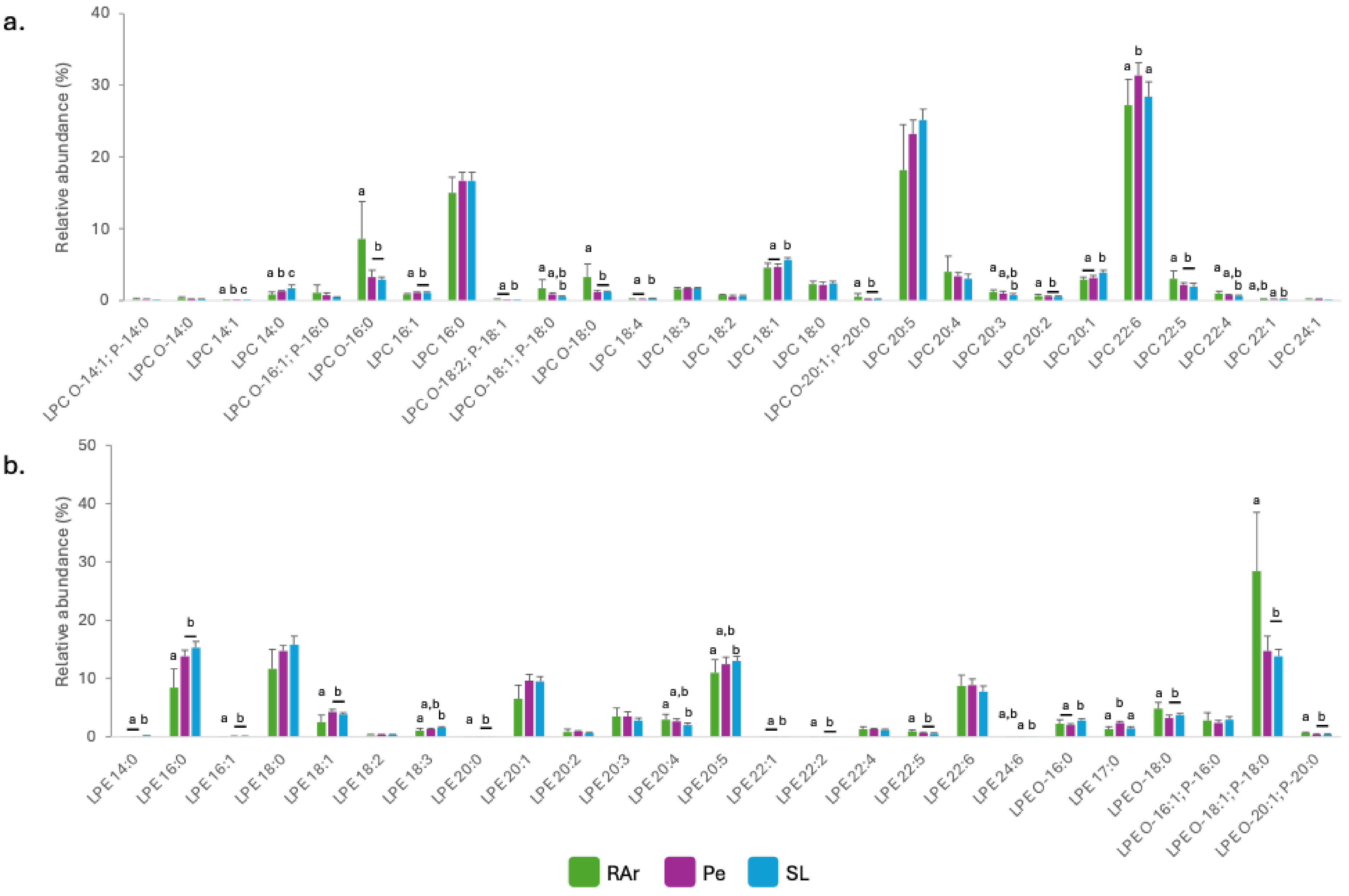

Supplementary Figure S3. Relative abundance of phospholipid species of a) lyso phosphatidylcholine (LPC) and b) lyso phosphatidylethanolamine (LPE) identified in common octopus' (*Octopus vulgaris*) arm muscle captured in three locations along the Iberian Atlantic coast (Ria Arousa – RAr, Peniche – Pe, and Santa Luzia – SL). Data represent relative abundance within the lipid class. Different letters indicate significant differences between sampling locations (Tukey's HSD post hoc test, $p < 0.05$).

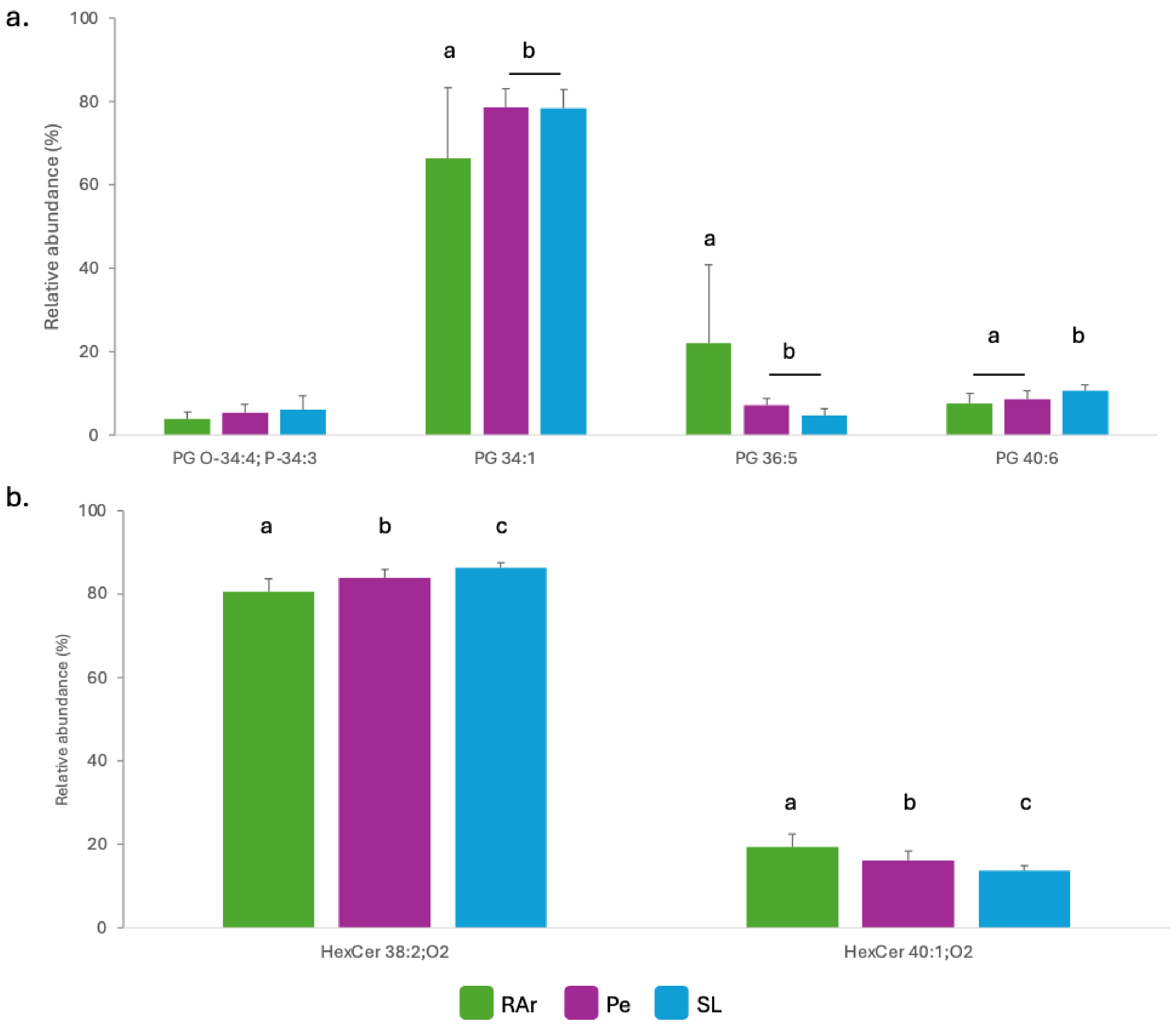

Supplementary Figure S4. Relative abundance of lipid species of a) phosphatidylglycerol (PG) and b) hexosylceramide (HexCer) identified in common octopus' (Octopus vulgaris) arm muscle captured in three locations along the Iberian Atlantic coast (Ria Arousa – RAr, Peniche – Pe, and Santa Luzia – SL). Data represent relative abundance within the lipid class. Different letters indicate significant differences between sampling locations (Tukey's HSD post hoc test, $p < 0.05$).

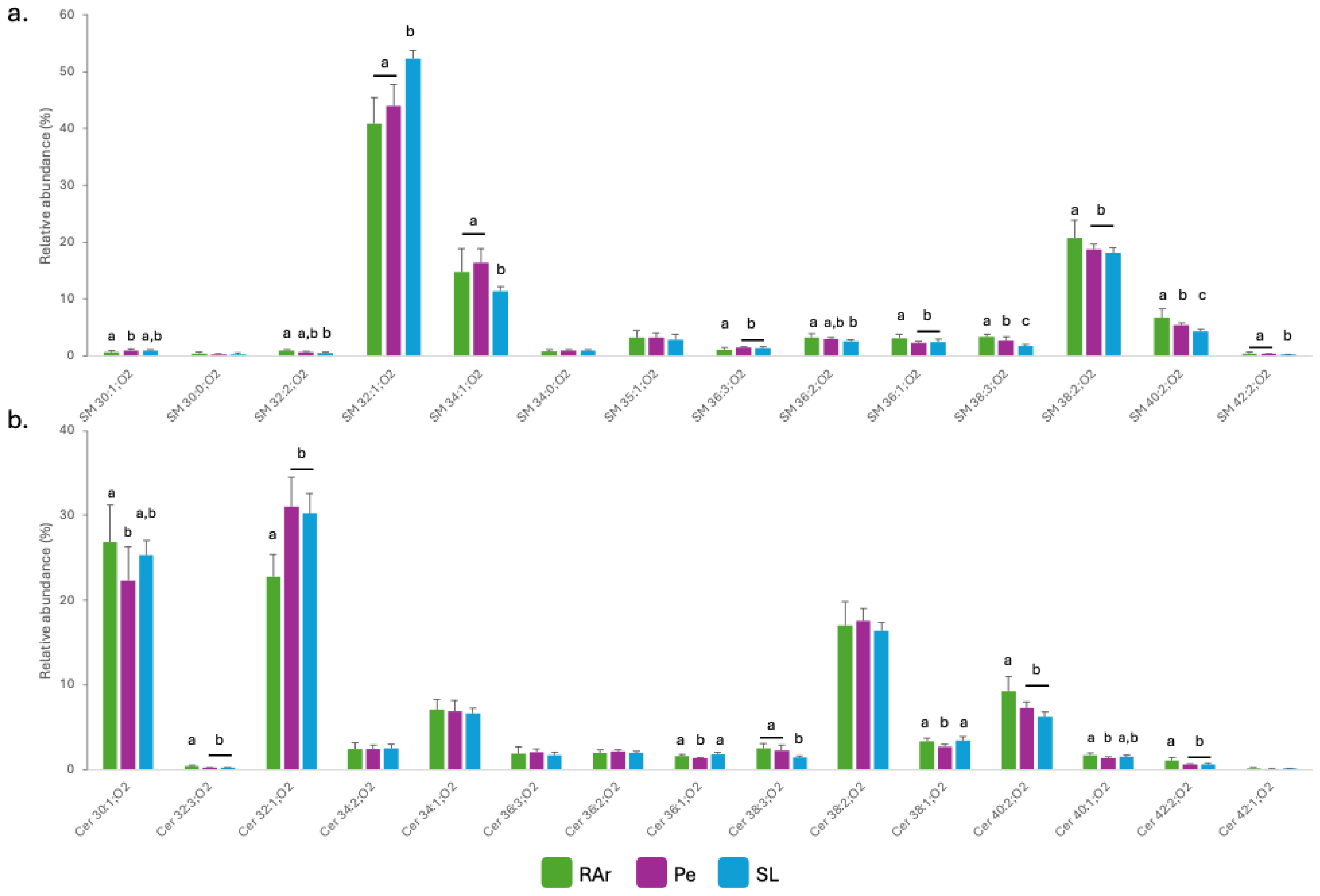

Supplementary Figure S5. Relative abundance of lipid species of a) ceramide (Cer) and b) sphingomyelin (SM) identified in common octopus' (*Octopus vulgaris*) arm muscle captured in three locations along the Iberian Atlantic coast (Ria Arousa – RAr, Peniche – Pe, and Santa Luzia – SL). Data represent relative abundance within the lipid class. Different letters indicate significant differences between sampling locations (Tukey's HSD post hoc test, $p < 0.05$).
